# IL-10/β-Endorphin-Mediated Neuroimmune Modulation on Microglia during Antinociception

**DOI:** 10.3390/brainsci13050789

**Published:** 2023-05-12

**Authors:** Thiago Caetano Andrade Belo, Gabriela Xavier Santos, Bruno Eduardo Gabriel da Silva, Bruno Lopes Gonçalves Rocha, Dennis William Abdala, Larissa Alves Moreira Freire, Fernanda Santos Rocha, Giovane Galdino

**Affiliations:** 1 Laboratory of Molecular Biology of Microorganisms, Federal University of Alfenas, Alfenas 37130-001, Brazil; thiago.belo@sou.unifal-mg.edu.br; 2 Laboratory of Neuroimmunobiology of Pain, Federal University of Alfenas, Alfenas 37130-001, Brazil; gabi_xaviersantos@yahoo.com.br (G.X.S.); brunoegs7@gmail.com (B.E.G.d.S.); bruno_lr@outlook.com (B.L.G.R.); fernandasantos.rocha@sou.unifal-mg.edu.br (F.S.R.); 3 Laboratory of Movement Analysis, Federal University of Alfenas, Alfenas 37130-001, Brazil; dennis.abdala@unifal-mg.edu.br; 4 Laboratory of Neuroscience, Neuroimmunomodulation and Pain Study, Federal University of Alfenas, Alfenas 37130-001, Brazil; larissaalves.freire@sou.unifal-mg.edu.br

**Keywords:** Interleukin 10, β-endorphin, microglia, pain, IL-10

## Abstract

Microglia are glial cells centrally related to pathophysiology and neuroimmunological regulation of pain through microglia–neuron crosstalk mechanisms. In contrast, anti-inflammatory mechanisms guided by immunological effectors such as IL-10 trigger the secretion of analgesic substances, culminating in the differential expression of genes encoding endogenous opioid peptides, especially β-endorphin. Thus, when β-endorphin binds to the µ-opioid receptor, it generates neuronal hyperpolarization, inhibiting nociceptive stimuli. This review aimed to summarize the recent advances in understanding the mechanism by which IL-10/β-endorphin can reduce pain. For this, databases were searched for articles from their inception up until November 2022. Two independent reviewers extracted the data and assessed the methodological quality of the included studies, and seventeen studies were considered eligible for this review. Several studies have demonstrated the impact of IL-10/β-endorphin in reducing pain, where IL-10 can stimulate GLP-1R, GRP40, and α7nAChR receptors, as well as intracellular signaling pathways, such as STAT3, resulting in increased β-endorphin expression and secretion. In addition, molecules such as gabapentinoids, thalidomide, cynandione A, morroniside, lemairamin, and cinobufagin, as well as non-pharmacological treatments such as electroacupuncture, reduce pain through IL-10 mediated mechanisms, reflecting a microglia-dependent β-endorphin differential increase. This process represents a cornerstone in pain neuroimmunology knowledge, and the results obtained by different studies about the theme are presented in this review.

## 1. Introduction

The global prevalence of pain standardized by age and sex is approximately 27.5%, ranging from 9.9% to 50.3% [1]. Although pain is a physiological mechanism important to protect and alert to the occurrence of real or potential damage, in some circumstances this pain can become pathological due to its chronic maintenance [2]. According to the International Association for the Study of Pain (IASP), chronic pain is considered a disease when it persists for more than three months [3]. In addition to the biological changes that chronic pain promotes, it drastically affects the quality of life, reflecting on social and psycho-emotional aspects [4,5].

Nociceptors are peripheral sensory neurons specialized in perceiving pain from chemical, mechanical, or thermal nociceptive stimuli [6]. The cell bodies of these neurons are located in the dorsal root ganglia (DRG) for the body and in the trigeminal ganglion for the face. They have a peripheral and a central axonal branch that, respectively, innervates their target organs and the spinal cord [7]. When the stimulus intensity reaches the noxious range, the nociceptors are excited, indicating that they have biophysical and molecular properties to detect and respond to potentially injurious stimuli [6,8,9]. The speed of impulse transmission through nociceptors will depend on their thickness and whether they are myelinated or not [6]. Two main classes of nociceptors are recognized: Type Aδ fibers (myelinated fibers with 2–5 μm diameter and up to 30 m/s conduction velocity) are involved in the transmission of intense short-term mechanical stimuli known as “first pain.” In addition, Type C fibers (unmyelinated fibers with less than 2 μm diameter and conduction velocity under 2 m/s) transmit diffuse signals of “second pain,” which is characterized by a dull pain and prolonged burning sensation [9,10]. Upon nociceptive stimulus detection by nociceptors, it is transmitted to the dorsal horn of the spinal cord, where it forms the first synapse with second-order neurons [6]. Central pain sensitization is a critical process in pain physiology, which will be addressed in this review. In pathological conditions like neuropathic pain, the synapse is potentiated due to the release of several neurotransmitters, leading to central pain sensitization [11].

Following the synapse, the second-order neuron conveys the nociceptive impulse via tracts and pathways to the reticular formation of the brainstem and thalamus [10]. At these sites, a second synapse takes place with third-order neurons, which transmit the stimulus to the somatosensory cortex [12]. Here, the response is processed and interpreted as pain [12].

As described above, during central pain sensitization, several neurotransmitters are released. Initially, it was believed that neurons were the primary sites of release, but recent studies have highlighted the crucial role of glial cells in this process. Glial cells, such as oligodendrocytes, microglia, and astrocytes, are now recognized as important players in the release of certain neurotransmitters and signaling molecules [13]. Oligodendrocytes are essential for axonal myelination in the central nervous system (CNS), playing a critical role in maintaining the integrity and function of neuronal circuits [14]. The full extent of the glial cell’s contribution to the modulation of neuronal function is still a relatively new area of investigation. Microglia, which constitute 5–12% of all CNS cells, are a diverse group of cells that share a mesodermal lineage with macrophages and are not yet fully understood in terms of their role in the CNS [15]. Astrocytes are the most numerous glial cells in the CNS, constituting about 19–40% of all glial cells, and providing crucial structural and metabolic support for neurons to facilitate neurotransmission [16,17].

Of all glial cells, microglia are considered the main cells involved in central pain sensitization, and their activation is triggered by the release of a range of inflammatory mediators and extracellular proteases derived from sensory neurons [18,19]. In addition, microglia can modulate the pain response depending on the activated phenotype, whether pro-inflammatory (M1) or anti-inflammatory (M2) [20]. The M1 polarization positively regulates genes encoding pro-inflammatory cytokines (especially TNF-α, IL-1β, IL-6, and IL-12), chemokines, nitric oxide, and brain-derived neurotrophic factor (BDNF) [20,21]. Conversely, the M2 phenotype activates anti-inflammatory factors, such as IL-4, arginase 1, and interleukin 10 (IL-10), with IL-10 being a classic marker of this phenotype [20,22]. Activated microglia can engage in crosstalk with astrocytes to exacerbate central pain sensitization, regardless of their phenotypic state [23]. This interaction involves the release of signaling molecules that can activate or inhibit astrocytes, which can reciprocally modulate microglial activity [23]. Therefore, microglia and astrocytes are crucial to studying the mechanisms underlying pain generation [23].

IL-10 acts as an immunomodulator in anti-inflammatory processes. It is mainly produced and released in peripheral tissues by immune cells and can also be produced by other cells, such as astrocytes and microglia in the central nervous system, acting on specific receptors expressed in dorsal root ganglion (DRG) neurons, peripheral nerves, and in the CNS, particularly in microglia and second-order neurons [24,25,26].

Initially identified as an endogenous hormone with morphine-like properties, β-endorphin is produced from the precursor molecule proopiomelanocortin (POMC) and exhibits a broad spectrum of biological activities, including its primary analgesic function [27]. In addition, β-endorphin is released in response to stressful and painful events, and its activity is primarily inhibitory [28]. At the peripheral level, β-endorphin has been shown to inhibit nociceptors depolarization, while inhibiting nociceptive transmission at the spinal and supraspinal levels [27]. Recent studies have shown a strong association between β-endorphin and IL-10 levels in the antinociceptive process, in addition to the previously established mechanisms of the peptide’s analgesic effect. These new findings will be the main focus of this review.

Based on recent evidence that IL-10 regulates neuroinflammatory processes in nervous system regions relevant to pain reduction, studies have demonstrated its ability to control proinflammatory cellular products that act to increase pain transmission [29]. Phosphorylation of transcription activator 3 (STAT3) upon activation of the IL-10 receptor on the microglial membrane is an important intracellular signaling pathway that downregulates pro-inflammatory substances. Accordingly, STAT3 recruitment upon Janus kinase activation encodes Bcl3 and SOCS3 expression, inhibiting the NF-κB pathway responsible for activating pro-inflammatory cytokine genes [24,29,30].

**Figure 1:**
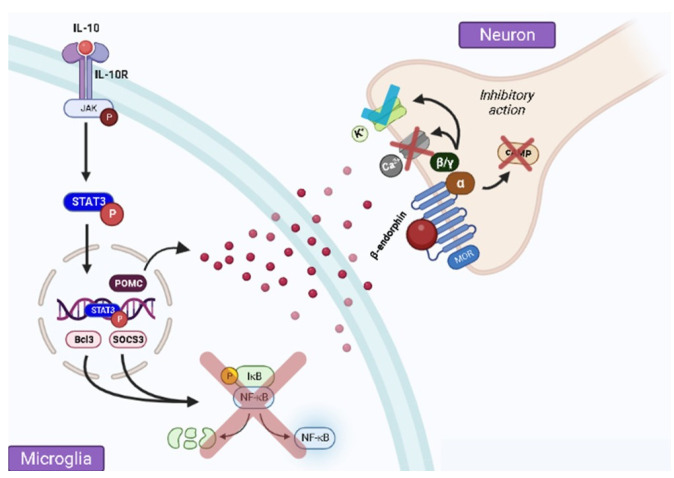
IL-10 mediates POMC expression and β-endorphin release, which then acts as a ligand on the MOR coupled to the G protein present in the neuronal membrane. This leads to the dissociation into subunits Gα and Gβγ inhibitory subunits. Gα subunit inhibits adenylate cyclase, while Gβγ subunit inhibits voltage-gated calcium channels (VGCC) and activates G protein-coupled inwardly rectifying potassium (GIRK) channels. This process ultimately results in the suppression of neurotransmission. The figure was created by BioRender.

Furthermore, IL-10-dependent STAT3 phosphorylation culminates in the expression of POMC and its conversion into β-endorphin, which acts by decreasing the nociceptive stimulus [30,31]. This process is due to the β-endorphin binding to the μ-opioid receptor (MOR), which is found in the neuronal membrane coupled to an inhibitory G protein [31]. Once activated, G protein subunits dissociate from receptors and act in different intracellular effector pathways that can lead to the suppression of neural functions [32,33]. The Gα subunit inhibits adenylyl cyclase, while Gβγ inhibits the opening of voltage-gated calcium channels (VGCC) and activates G-protein-activated potassium channels (GIRK) postsynaptically, resulting in reduced neurotransmitter release and membrane hyperpolarization, as shown in Figure 1 [33].

In this review, we will demonstrate the role of spinal microglia in IL-10 and β-endorphin-mediated pain modulation. We will highlight IL-10’s importance, which is an anti-inflammatory cytokine that controls pain. Additionally, we will provide evidence that IL-10 can stimulate the release of other pain-relieving substances, such as β-endorphin. The synergistic action between IL-10 and β-endorphin helps to control nociceptive transmission, especially in the spinal cord.

After verifying a correlation between IL-10 levels in the differential expression of β-endorphin encoding genes and its antinociceptive action, this review aimed to address the studies that evaluated IL-10/β-endorphin role in pain neuroimmunomodulation, indicating pathways and receptors that may be related to pain reduction via IL-10/β-endorphin. Furthermore, the influence of pharmacological and non-pharmacological antinociceptive therapies on IL-10/β-endorphin activation was evaluated.

## 2. Pain Control by IL-10 via β-Endorphin Release at the Spinal Level

**Figure 2:**
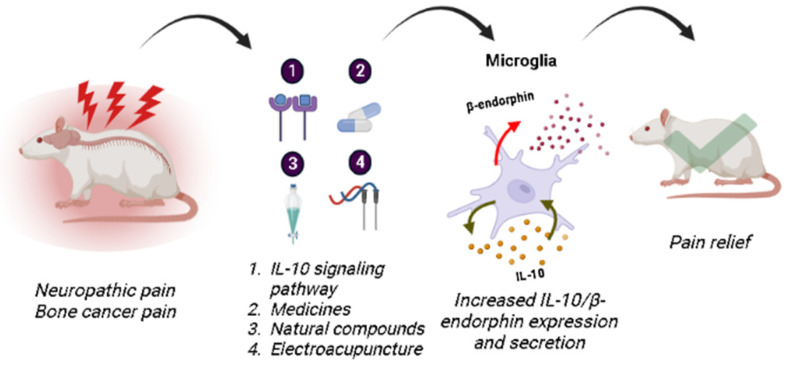
Animal models of neuropathic pain and bone cancer pain, treated with various approaches (including the activation of specific receptors such as GPR40 and GLP-1R, drugs such as thalidomide and gabapentinoids, natural compounds such as cynandione, morroniside, lemairamin, and cinobufagin, and non-pharmacological treatments such as electroacupuncture) increased the expression and secretion of IL-10/β-endorphin. This increase led to a reduction in mechanical allodynia and hyperalgesia. The figure was created by BioRender.

Recent evidence has demonstrated IL-10 and β-endorphin impact on pain control [30,34,35,36,37,38,39,40,41,42,43]. Thus, studies analyzing these two protagonists were selected, in which microglia proved to be the main cell involved in this process, as shown in Table 1, Figure 2, and the graphical abstract.

In experimental models of neuropathic pain such as spinal nerve ligation, mechanical allodynia accompanied by increased levels of inflammatory markers is verified without changes in IL-10 and β-endorphin spinal levels [31,34]. However, intrathecal IL-10 administration in mice with neuropathic pain promoted antiallodynic and antihyperalgesic effects. In this model, reduced levels of the proinflammatory cytokines TNF-α, IL-1β and IL-6 levels were identified; as well as lower neuronal amplitude and frequency, which are usually high during neuropathic pain [30,35]. The antinociceptive effect found after IL-10 administration has been attributed to increased POMC and β-endorphin levels, since intrathecal administration of β-endorphin neutralizing antibodies and MOR antagonists blocked the IL-10-induced antiallodynic effect [30]. Furthermore, these effects were confirmed by in vitro assays, which found IL-10-induced β-endorphin release by microglial cells, but not in astrocytes or neurons. In addition, these authors showed that when microglia was inhibited before IL-10 stimulation, POMC and β-endorphin levels were not altered [30].

IL-10 downregulates the pro-inflammatory cytokines TNF-α, IL-1β, and IL-6 by inducing SOCS3 and Bcl3 gene expression, which in turn inhibit the NF-κB pathway [29,30]. This mechanism has been proposed to explain IL-10-induced anti-inflammatory effects. However, pro-inflammatory substance reduction was not associated with an increased nociceptive threshold in mice with neuropathic pain, with this antiallodynic effect attributed to increased β-endorphin levels after IL-10 stimulation [30].

## 3. Spinal Microglial Activation Increases IL-10/β-Endorphin Levels

Peripheral or central nerve injuries usually course with maladaptive neuroplasticity due to a central sensitization process in the spinal cord by hyperactivity in primary afferent neurons or glial cells [8]. Increased IL-10 and β-endorphin spinal cord levels seem to attenuate this hyperexcitability [35]. Furthermore, spinal activation of glucagon-like peptide-1 receptor (GLP-1R), G protein-coupled receptor 40 (GPR40), and alpha-7 nicotinic acetylcholine receptor (α7-nAChR) has been shown to elevate IL-10 and β-endorphin levels, resulting in an antinociceptive effect [31,36,37,38,39,40].

The antinociceptive effect promoted by GLP-1R activation by exenatide or morronoside agonists was associated with increased IL-10, β-endorphin and POMC gene expression [31,36]. Opposite results were observed after the administration of anti-IL-10 and anti-β-endorphin neutralizing antibodies, abolishing exenatide- and morronoside-induced antinociception [31,36]. The underlying mechanisms that led to IL-10/β-endorphin upregulation upon GLP-1R activation in microglia were investigated in vitro. Accordingly, it has been demonstrated that increased IL-10 gene expression after exenatide administration is mediated by the cAMP/PKA/p38/CREB pathway [31]. Similar results were observed in mice, where the underlying mechanism of IL-10 release via α7-nAChR activation by cynandione also occurred through the cAMP/PKA/p38/CREB pathway [37]. IL-10 acts autocrinely when released by microglia, activating STAT3, which induces β-endorphin gene expression [31,36,37].

Like GLP-1R, α7-nAChR activation by cynandione A, lemairamin, and cinobufagin also increased IL-10 and POMC gene expression, stimulating IL-10/β-endorphin secretion by microglia in animal models of neuropathic and bone cancer pain, resulting in pain reduction through antiallodynic behavior, as verified by the von Frey filament test [37,38,39]. In addition, α7-nAChR activation by cynandione A, GPR40, and GW9508 share the p38/JNK/ERK intracellular signaling pathway for encoding IL-10 in microglia [37,40]. Thus, microglia seem to be the main cells involved in IL-10/β-endorphin secretion [37,40].

Thalidomide, an anti-inflammatory and immunomodulatory drug with teratogenic effects, also has impacts on the IL-10/β-endorphin signaling pathway [44]. Deng and colleagues [41] verified that this drug relieves pain sensation by suppressing pro-inflammatory cytokine production in microglia and stimulating IL-10/β-endorphin expression. Blocking IL-10 action before the thalidomide treatment inhibited the β-endorphin encoding gene, in addition to not suppressing genes encoding pro-inflammatory cytokines in microglia cultures, showing that thalidomide decreases neuropathic pain by upregulating IL-10/β-endorphin expression in microglia [41].

A study conducted by Ahmad and colleagues [42] also found similar results using gabapentinoids, which comprise a group of drugs widely used to treat neuropathic pain, as they have the ability to bind to the calcium channel α2δ-1 subunit in voltage-dependent neurons [45,46]. Thus, these authors demonstrated that gabapentinoid intrathecal injection inhibited mechanical allodynia, and this effect was associated with increased IL-10 and β-endorphin gene expression in the spinal cord of rats with neuropathic pain. Moreover, this process was reversed after microglia inhibition, thus demonstrating that gabapentinoid-induced antinociception is mediated by IL-10/β-endorphin release.

Electroacupuncture is a well-recognized non-pharmacological treatment for chronic pain control in humans and animals [47]. Ali and colleagues [43] found that low-frequency electroacupuncture stimulation promoted neuropathic pain relief in neuropathic rats in a time-dependent manner, increasing IL-10 and β-endorphin gene expression in spinal microglia. Furthermore, anti-IL-10 pre-treatment blocked β-endorphin gene expression, thus showing the role of IL-10/β-endorphin in reducing pain during electroacupuncture stimulation [43].

## 4. β-Endorphin Does Not Participate in IL-10-Induced Antinociception at the Peripheral Level

A nociceptive paradox can be observed in experimental models of peripheral neuropathy [48]. Diabetic neuropathy, which affects the peripheral nerves, is a common complication in subjects with diabetes mellitus, and gabapentin has often been indicated as the treatment of choice to control the recurrent pain in this disease [49]. Interestingly, Abdel and colleagues [50] found reduced β-endorphin levels in rats with diabetic neuropathy, and this process was not restored even after gabapentin administration. Furthermore, increased IL-1β, IL-10, and TNF-α serum levels were identified, which were reversed after gabapentin treatment [50]. These findings reinforce the microglia’s role as responsible for IL-10/β-endorphin-dependent pain modulation at the spinal level, since increased β-endorphin expression accompanied by increased IL-10 spinal cord levels was specifically observed in microglial cells and does not transpose to the peripheral level in this case.

Furthermore, studies have verified that increased IL-10 expression does not culminate in greater peripheral β-endorphin expression [51,52]. Met-RANTES is a selective CCL5 antagonist, which correlates with pain-generating mechanisms, decreasing inflammatory and nociceptive responses. Liou and colleagues [51] evaluated Met-RANTES intraperitoneal administration in mice with neuropathic pain, and found that this treatment reduced mechanical allodynia after sciatic nerve ligation, increasing IL-10 and reducing macrophage infiltration and β-endorphin expression [51]. Increased IL-10 levels accompanied by reduced macrophage infiltration would explain the impossibility of IL-10-induced β-endorphin expression, since the same group previously showed that opioid peptides are initially secreted by neutrophils and later by macrophages [51,53,54].

Another study conducted by Leiguarda and colleagues [52] also associated reduced immune cells (e.g., macrophages) with lower β-endorphin expression in the inflammatory infiltrate, even in the presence of IL-10 in an inflammatory pain model induced by complete Freund’s adjuvant and the impact of IMT504 treatment in mice, a PyNTTTTGT family oligodeoxynucleotide. IMT504 treatment had an antiallodynic effect, resulting from the modulation of the inflammatory environment, since pro-inflammatory cytokines and chemokines were downregulated, and IL-10 was upregulated. However, this antinociceptive effect was not related to β-endorphin once its levels were reduced [52].

Interestingly, Mannaa and colleagues [55] found hypoalgesia (increased nociceptive threshold), as evaluated by the hot plate test in animals with dichloroacetate-induced peripheral neuropathy, and this effect was associated with increased IL-10 and β-endorphin serum levels. During neuroinflammation, analgesic and anti-inflammatory pathways can be activated in an attempt to modulate the response. In addition, the authors of this previous study demonstrated reduced pain sensitivity with gabapentin-treatment or non-pharmacological approaches based on low-level laser therapy (LLLT), to which IL-10/β-endorphin may be related [55]. Further studies are needed to assess its effects at peripheral levels.

Non-pharmacological therapies, such as transcranial direct current stimulation (tDCS), have been growing in recent years for pain control. tDCS has been considered a promising non-pharmacological therapy for chronic pain treatment. To investigate the tDCS mechanisms, a clinical study was carried out with patients diagnosed with knee osteoarthritis who received tDCS treatment [56]. After treatment, reduced IL-6, TNF-α, β-endorphin, and IL-10 levels were observed, suggesting therapy efficiency in controlling inflammation, but the study did not clarify a potential IL-10/β-endorphin impact in this condition [56]. However, IL-10/β-endorphin involvement cannot be completely ruled out, since the authors investigated only IL-10/β-endorphin circulating levels, requiring experimental studies to investigate IL-10/β-endorphin spinal cord levels after tDCS treatment [56].

Furthermore, a theme that has been growing in recent years is how immunometabolic processes act on the functionality of the immunological component. Sun and colleagues [57] checked that itaconate, a metabolite arising from the tricarboxylic acid cycle (TCA) [58] commonly secreted after macrophage activation through the enzyme cis-aconitate decarboxylase 1 (IRG1), can mediate the analgesic effect in chronic constrictive nerve injury (CCI) mouse models via IL-10/STAT3/β-endorphin. IRG1 deficient mice showed greater mechanical and heat hypersensitivity. In this model, neuropathic pain was relieved after itaconate administration. Interestingly, this effect was impaired in IL-10 knockout mice, demonstrating that itaconate treatment increased IL-10 and β-endorphin levels, which were especially secreted by spinal neurons, a process whose microglia involvement still needs to be investigated [57]. Thus, approaches that assess the immunometabolic impact on microglia and its reflection on IL-10/β-endorphin-mediated neuroimmune modulation may become a key point to elucidate and propose new studies and therapeutic models aimed at combating pain.

## 5. Future Perspectives

This review emphasizes the crucial role of the IL-10/β-endorphin pathway in the neuroimmunomodulation of pain, particularly at the microglia-dependent spinal level. However, the existing literature provides limited evidence that extensively demonstrates its role, as studies have only focused on a narrow range of stimulant molecules or treatments where IL-10/β-endorphin has been identified as a key target in reducing pain. Thus, this review can provide a basis for future pain studies to investigate the involvement of the IL-10/β-endorphin pathway in their experimental models. Moreover, the impact of this pathway in combating pain at the peripheral level remains uncertain, and further detailed investigations are necessary to confirm or refute its involvement in other anatomical sites. Nonetheless, the IL-10/β-endorphin pathway holds potential as a target for pre-clinical and clinical studies aimed at reducing pain through specific therapies that enhance the secretion of IL-10/β-endorphin at the spinal level.

## 6. Conclusions

The studies addressed in this review indicated that IL-10 is required for differential β-endorphin gene expression by microglia, stimulating its subsequent release into the spinal cord extracellular environment, thus leading to antinociception. Furthermore, this study presented several molecular mechanisms involved in IL-10/β-endorphin-induced antinociception, such as GLP-1R, GPR40, and α7nAChR. The impact of pharmacological interventions on this pathway was another important point demonstrated. Antinociceptive effects produced by gabapentinoids and thalidomide; natural compounds such as cynandione A, morronoside, lemairamin, and cinobufagin; as well as non-drug treatments such as electroacupuncture, may be directly associated with IL-10/β-endorphin pathway activation. Taken together, the findings of this review shed light on the interaction between IL-10/β-endorphin, and the mechanisms involved in pain control, which may serve as a starting point for several existing studies that evaluate the antinociceptive effects of pharmacological or non-pharmacological therapies or future studies of new compounds that show to be promising antinociceptive candidates. However, further studies are needed, mainly clinical trials that assess the involvement of the IL-10/β-endorphin pathway in different types of pain, so that research investigating treatments that act on these mechanisms can be initiated, since the present review addressed only preclinical trials.

## Figures and Tables

**Table 1:** Microglial mechanisms involved in the increase of IL-10/ β-endorphin in the pain decrease.

	Increased IL-10/β-Endorphin Signaling in Microglia	IL-10/β-Endorphin Impact on Pain Decrease	Refs.
IL-10 signaling pathway	**GLP-1 R activation by exenatin/IL-10:** GLP-1 activation inhibits glutamatergic transmission and pain hypersensitivity via β-endorphin MOR for IL-10 signaling	↓ mechanical allodynia and thermal hyperalgesia in neuropathic rats ↓ excitatory postsynaptic currents dorsal horn neurons of lamina II	[34,35]
	**GPR40 receptor activation by the GW9508 agonist:** ↑ expression of IL-10/β-endorphin genes and proteins	↓ mechanical allodynia and hyperalgesia in rats with neuropathic pain	[40]
	**Administration of intrathecal IL-10**: ↑ β-endorphin expression ↑ POMC mRNA expression ↑ pSTAT3	↓ mechanical allodynia and thermal hyperalgesia in male and female neuropathic rats	[31]
Medicines	**Gabapentinoids:** ↑IL-10/β-endorphin mRNA expression	↓ mechanical allodynia of rats with neuropathic pain	[42]
	**Thalidomide:** ↑ IL-10/β-endorphin expression	↓ mechanical allodynia and thermal hyperalgesia in rats with neuropathic pain	[41]
Natural compounds	**α7-nAChR receptor activation by Cynandione A:** ↑ IL-10/POMC mRNA expression ↑ IL-10/β-endorphin protein expression ↑ pSTAT3 ↑ pPKA, p38, and CREB	↓ mechanical allodynia in neuropathic pain rats	[37]
	**GLP-1R Receptor Activation by Morroniside:** ↑ IL-10/β-endorphin/POMC gene expression	↓ mechanical allodynia in neuropathic pain rats	[36]
	**α7-nAChRs receptor activation by Lemairamin:** ↑ IL-10/β-endorphin protein expression ↑ IL-10 mRNA expression	↓ mechanical allodynia of rats with neuropathic pain and bone cancer pain	[38]
	**α7-nAChRs receptor activation by Cinobufagin:** ↑ IL-10/POMC mRNA expression ↑ IL-10/β-endorphin protein expression	↓ mechanical allodynia of rats with bone cancer pain	[39]
Non-pharmacological treatment	**Electroacupuncture:** ↑ IL-10/β-endorphin gene and protein expression ↑ POMC gene expression	↓ mechanical allodynia of rats with neuropathic pain	[43]

IL-10 is the abbreviation for interleukin 10; GLP-1 is the abbreviation for glucagon-like peptide-1; MOR is the abbreviation for μ-opioid receptor; GPR 40 is the abbreviation for G protein-coupled receptor 40; POMC is the abbreviation for precursor molecule proopiomelanocortin; pSTAT3 is the abbreviation for phosphorylated transcription activator 3; mRNA is the abbreviation for messenger ribonucleic acid; pPKA is the abbreviation for phosphorylated protein kinase A; CREB is the abbreviation for cAMP-responsive element binding protein; α7-nAChR is the abbreviation for alpha7 nicotinic acetylcholine receptor. (↑) up arrows indicate increase and (↓) down arrow indicates decrease.

## Data Availability

The data presented in this study are available on request from the corresponding author.

## Abbreviations

α7-nAChR, alpha7 nicotinic acetylcholine receptor; Bcl3, B-cell lymphoma 3; BDNF, brain-derived neurotrophic factor; CREB, cAMP-responsive element binding protein; CCL5, C-C motif chemokine ligand 5; CNS, central nervous system; cAMP, cyclic adenosine monophosphate; DRG, dorsal root ganglia; GPR 40, G protein-coupled receptor 40; GLP-1, glucagon-like peptide-1; GLP-1R, glucagon-like peptide-1 receptor; GIRK, G-protein-activated potassium channel; IRG1, immune-Responsive Gene 1; IL-1β, interleukin- 1 beta; IL-10, interleukin- 10; IL-12, interleukin- 12; IL-4, interleukin- 4; IL-6, interleukin- 6; IASP, International Association for the Study of Pain; LLLT, Low-level laser therapy; mRNA, messenger ribonucleic acid; NF- κB, nuclear factor kappa-light-chain-enhancer of activated B cells; POMC, precursor molecule proopiomelanocortin; PKA, protein kinase A; SOCS3, suppressor of cytokine signaling 3; tDCS, transcranial direct current stimulation; STAT3, transcription activator 3; TCA, tricarboxylic acid cycle; TNF-α, tumor necrosis factor alpha; VGCC, voltage-gated calcium channels; MOR, μ-opioid receptor.
